# Supplementary material for: Salmonella enterica Serovar Dublin from Cattle in California from 1993–2019: Antimicrobial Resistance Trends of Clinical Relevance
Source: Antibiotics (Basel). 2022 Aug 17;11(8):1110. doi: 10.3390/antibiotics11081110 (PMC9405026; doi:10.3390/antibiotics11081110)
Supplement: Supplementary file 1 [file antibiotics-11-01110-s001.zip › antibiotics-1831256-supplementary.pdf]

Supplemental Table S1a. Variables removed from the final model that classified isolates as resistant for cephalosporin drugs in the NARMS panel. For results from final model refer to Table 4b.

Variable[1]	Ceftiofur		Ceftriaxone		Cefoxitin	
	OR[2]	P value[4]	OR[2]	P value[4]	OR[2]	P value[4]
**Age Group[5]**		0.39		0.39		0.49
**Cow vs Early PW**	0.6	0.40	0.5	0.39	0.6	0.5
**Cow vs Early HF**	1.2	0.78	1.2	0.78	1.2	0.79
**Cow vs Late PW**	0.8	0.79	0.8	0.78	0.7	0.60
**Early PW vs Early HF**	2.1	0.10	2.1	0.10	1.9	0.14
**Early PW vs Late PW**	1.4	0.40	1.4	0.40	1.1	0.80
**Early HF vs Late PW**	0.7	0.45	0.7	0.45	0.5	0.28
**Season[6]**		0.15		0.14		0.45
**Fall vs Spring**	2.4	0.09	2.4	0.09	1.4	0.46
**Fall vs Summer**	2.5	0.05	2.5	0.05	2.1	0.11
**Fall vs Winter**	1.2	0.63	1.2	0.63	1.3	0.59
**Spring vs Summer**	1.0	0.91	1.0	0.91	1.5	0.44
**Spring vs Winter**	0.5	0.20	0.5	0.21	0.9	0.82
**Summer vs Winter**	0.5	0.13	0.5	0.13	0.6	0.28

1. Pairwise odds ratio comparison for all response variable levels. The first response level is the reference (e.g. if OR is equal to 0.5 for year group 1993-99 vs 2000-05, this means there is a 0.5 odds ratio for isolation of a *S.* Dublin resistant to the referred antimicrobial in the 1993-99 year interval when compared to the 2000-05 year interval).
2. Odds ratio.
3. This represents the 95% confidence interval of the odds ratio.
4. *P* value for the odds ratio.
5. Age groups of animals from which *S.* Dublin was isolated. Early PW (Early preweaned): ≤ then 4 weeks of age; Late PW (Late preweaned): > 4 weeks of age and ≤ 9 weeks of age; Early HF (Early Heifer): > 9 weeks of age and ≤ 12 months of age; Cow: > 17 months of age.
6. Season of the year when *S.* Dublin was isolated.

Supplemental Table S1b. Variables removed from the final model that classified isolates as resistant for aminoglycosides. For results from final model refer to Table 4b.

Variable[1]	Gentamicin		Streptomycin	
	OR[2]	P value[4]	OR[2]	P value[4]
**Age Group[5]**		0.40		0.11
**Cow vs Early PW**	0.3	0.09	0.3	0.04
**Cow vs Early HF**	0.3	0.15	0.2	0.03
**Cow vs Late PW**	0.3	0.12	0.5	0.26
**Early PW vs Early HF**	1.0	0.89	0.6	0.45
**Early PW vs Late PW**	1.0	0.94	1.6	0.32
**Early HF vs Late PW**	1.0	0.95	2.7	0.17
**Season[6]**		0.38		0.056
**Fall vs Spring**	1.0	0.97	4.1	0.02
**Fall vs Summer**	0.5	0.20	2.7	0.10
**Fall vs Winter**	0.5	0.24	1.2	0.80
**Spring vs Summer**	0.5	0.29	0.6	0.42
**Spring vs Winter**	0.5	0.26	0.3	0.03
**Summer vs Winter**	1.1	0.90	0.4	0.16

1. Pairwise odds ratio comparison for all response variable levels. The first response level is the reference (e.g. if OR is equal to 0.5 for year group 1993-99 vs 2000-05, this means there is a 0.5 odds ratio for isolation of a *S.* Dublin resistant to the referred antimicrobial in the 1993-99 year interval when compared to the 2000-05 year interval).
2. Odds ratio.
3. This represents the 95% confidence interval of the odds ratio.
4. *P* value for the odds ratio.
5. Age groups of animals from which *S.* Dublin was isolated. Early PW (Early preweaned): ≤ then 4 weeks of age; Late PW (Late preweaned): > 4 weeks of age and ≤ 9 weeks of age; Early HF (Early Heifer): > 9 weeks of age and ≤ 12 months of age; Cow: > 17 months of age.
6. Season of the year when *S.* Dublin was isolated.

Supplemental Table S1c. Variables removed from the final model that classified isolates as resistant for quinolones and aminopenicillins by antimicrobial class for antimicrobial drugs in the NARMS panel. For results from final model refer to Table 4c.

Variable[1]	Nalidixic Acid		Ampicillin		AMC*	
	**OR[2]**	***P* value[4]**	**OR[2]**	***P* value[4]**	**OR[2]**	***P* value[4]**
**Age Group[5]**		0.45		0.20		0.40
**Cow vs Early PW**	1.1	0.95	0.4	0.08	0.6	0.39
**Cow vs Early HF**	0.4	0.50	0.8	0.74	1.2	0.78
**Cow vs Late PW**	0.6	0.70	0.6	0.36	0.8	0.79
**Early PW vs Early HF**	0.4	0.12	2.1	0.09	2.1	0.10
**Early PW vs Late PW**	0.6	0.36	1.5	0.32	1.4	0.40
**Early HF vs Late PW**	1.4	0.58	0.7	0.48	0.7	0.45
**Season[6]**		0.72		0.06		0.14
**Fall vs Spring**	0.9	0.88	3.8	0.01	2.4	0.09
**Fall vs Summer**	1.5	0.55	2.9	0.03	2.5	0.05
**Fall vs Winter**	0.7	0.59	1.9	0.17	1.2	0.63
**Spring vs Summer**	1.6	0.49	0.7	0.55	1.0	0.91
**Spring vs Winter**	0.8	0.75	0.5	0.17	0.5	0.21
**Summer vs Winter**	0.5	0.25	0.7	0.38	0.5	0.13

1. Pairwise odds ratio comparison for all response variable levels. The first response level is the reference (e.g. if OR is equal to 0.5 for year group 1993-99 vs 2000-05, this means there is a 0.5 odds ratio for isolation of a *S.* Dublin resistant to the referred antimicrobial in the 1993-99 year interval when compared to the 2000-05 year interval).
2. Odds ratio.
3. This represents the 95% confidence interval of the odds ratio.
4. *P* value for the odds ratio.
5. Age groups of animals from which *S.* Dublin was isolated. Early PW (Early preweaned): ≤ then 4 weeks of age; Late PW (Late preweaned): > 4 weeks of age and ≤ 9 weeks of age; Early HF (Early Heifer): > 9 weeks of age and ≤ 12 months of age; Cow: > 17 months of age.
6. Season of the year when *S.* Dublin was isolated.
